# Supplementary material for: Metabolite Patterns in Human Myeloid Hematopoiesis Result from Lineage-Dependent Active Metabolic Pathways
Source: Int J Mol Sci. 2020 Aug 24;21(17):6092. doi: 10.3390/ijms21176092 (PMC7504406; doi:10.3390/ijms21176092)
Supplement: Supplementary file 1 [file ijms-21-06092-s001.zip › ijms-894011 supp for conversion/Supplement_Dei_Unique_Metabolites.pdf]

Supplementary Material

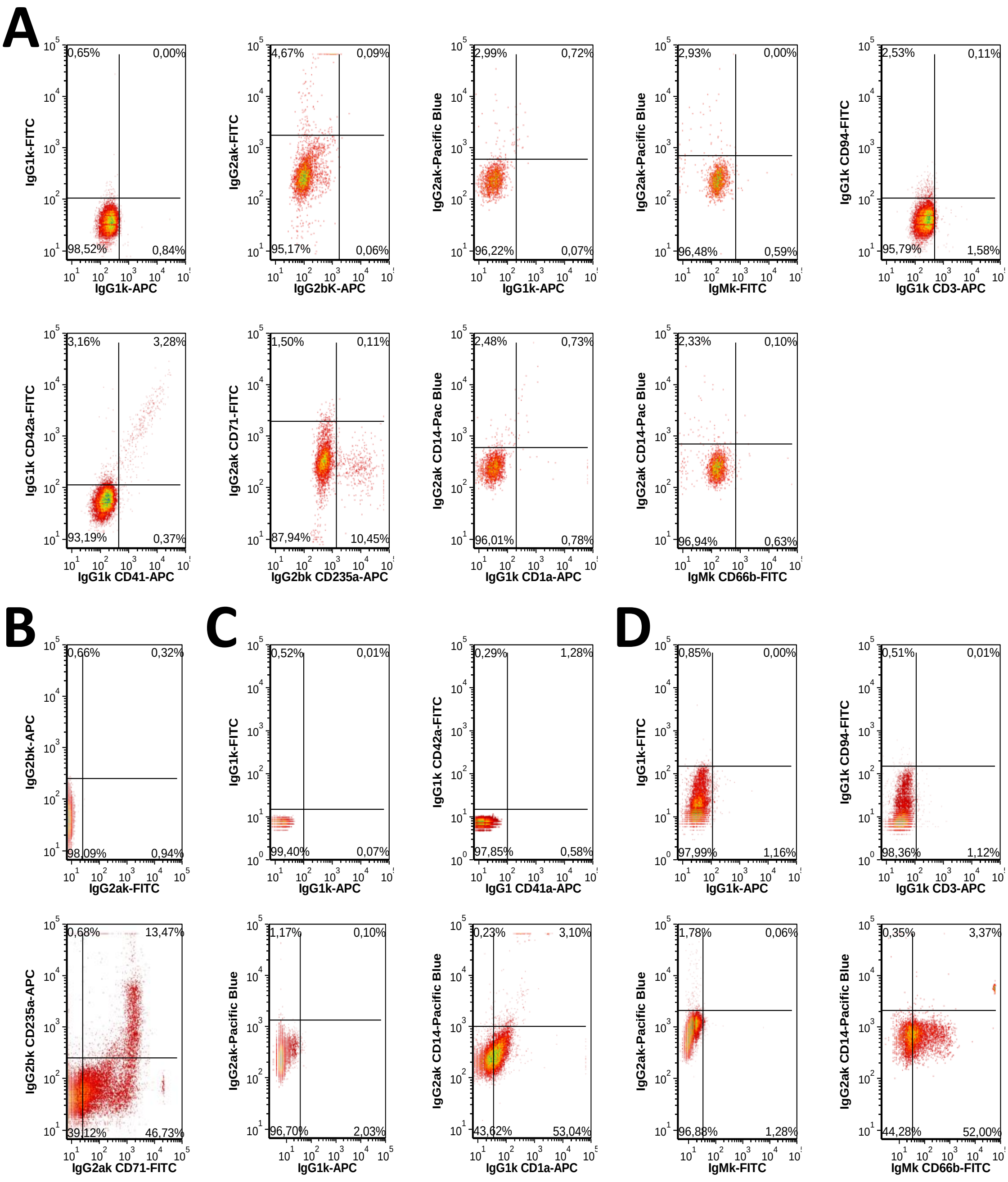

Figure S1. Expression of typical lineage markers in the different populations. (**A**) Expression of typical lineage selective surface markers in the progenitor population in comparison to corresponding isotype controls, determined by flow cytometry. Representative dot plots from three independent experiments (n = 3) are shown. (**B**) Expression of erythroid (CD71 and CD235a) lineage surface markers in the erythrocyte population in comparison to corresponding isotype controls, determined by flow cytometry. Representative dot plots from three independent experiments (n = 3) are shown. (**C**) Expression of megakaryocytic (CD41a, CD42a), monocytic (CD14) and dendritic cell / Langerhans cell (CD1a) lineage surface markers in the dendritic cell population in comparison to corresponding isotype controls, determined by flow cytometry. Representative dot plots from three independent experiments (n = 3) are shown. (**D**) Expression of natural killer T cell (CD3, CD94), monocytic (CD14) and granulocyte (CD66b) lineage surface markers in the neutrophil population in comparison to corresponding isotype controls, determined by flow cytometry. Representative dot plots from three independent experiments (n = 3) are shown.

Table S1. Set of expressed genes in each generated population. For each population, the four most dominant transcripts (marked in bold), in combination with a *q*-value < 0.05, were used for verification of major cell populations. Furthermore, several other dominant transcripts, with a *q*-value < 0.05 for the corresponding population and a *q*-value > 0.05 for the other populations are included.

gene	Protein	Erythrocyte	Dendritic cells	Neutrophils	Q erythrocyte	Q dendritic cells	Q neutrophils
ALCAM	CD166	23,502	108,142	21,096	0,995	**0,030**	0,992
AZU1	Azurocidin	384,931	200,726	**807,309**	0,026	0,127	0,026
BLVRB	Flavin reductase (NADPH)	276,353	112,461	82,272	**0,026**	0,989	0,992
C1QA	Complement C1q subcomponent subunit A	49,944	172,111	155,910	0,070	0,052	**0,026**
C1QB	Complement C1q subcomponent subunit B	44,129	249,230	226,499	0,087	0,030	0,026
C1QC	Complement C1q subcomponent subunit C	77,604	**348,741**	278,409	0,026	0,030	0,043
CTSG	Cathepsin G	126,910	73,336	313,856	0,026	0,765	0,026
DPP4	Dipeptidyl peptidase 4	15,285	68,023	9,535	0,087	**0,030**	0,581
ELANE	Neutrophil elastase	**756,959**	254,768	**1721,370**	0,026	0,194	0,026
EPX	Eosinophil peroxidase	95,303	**274,194**	1,284	0,026	0,030	0,992
FECH	Ferrochelatase, mitochondrial	67,223	17,773	20,084	**0,026**	0,989	0,992
GPNMB	Glycoprotein NMB	46.312	**751.746**	784.673	0.042	0.029	0.0256
GP5	CD42d	0,000	0,000	0,000	0,026	0,030	0,058
HBB	Hemoglobin subunit beta	**1538,550**	28,784	9,215	0,026	0,077	0,043
HBM	Hemoglobin subunit mu	60,432	0,658	0,421	**0,026**	0,359	0,708
ITGA2B	CD41	38,119	21,551	34,052	0,026	0,030	0,292
KEL	CD238	60,424	1,476	2,070	**0,026**	0,503	0,992
KLF1	Krueppel-like factor 1	89,940	1,970	2,159	**0,026**	0,184	0,276
MRC1	CD206	195,641	**506,575**	92,780	0,026	0,030	0,992
PRTN3	Myeloblastin	**732,813**	180,812	**1141,910**	0,026	0,941	0,026
RHAG	CD241	188,878	20,615	4,118	**0,026**	0,989	0,344
S100A8	Protein S100-A8	547,594	685,878	**1475,440**	0,078	0,094	0,026
SLC4A1	CD233	65,298	0,250	0,353	**0,026**	1,000	0,992
TFRC	CD71	**490,420**	131,349	85,104	0,026	0,989	0,992

Table S1. Significant metabolite changes, with an absolute $\log_2$ fold change > 1, during erythroid differentiation. Given are the $\log_2$ fold changes, compared to the progenitor population and the corresponding FDR adjusted *p*-value.

Metabolite	logFC	adj. p
PC aa C42:4	1.907	0.000
SM-OH C16:1	1.802	0.002
PC aa C40:3	1.769	0.000
Histamine	1.733	0.001
PC aa C28:1	1.723	0.001
lysoPC a C28:0	1.681	0.001
PC aa C40:2	1.656	0.000
PC aa C42:5	1.611	0.000
SM C16:0	1.576	0.005
PC aa C40:4	1.566	0.000
PE aa C40:3	1.554	0.000
PC aa C30:2	1.538	0.002
SM C18:0	1.522	0.005
SM C24:1	1.515	0.007
Putrescine	1.495	0.000
PC aa C34:1	1.490	0.005
lysoPC a C28:1	1.465	0.003
SM -OH C14:1	1.442	0.008
PC aa C32:0	1.441	0.008
PE aa C40:4	1.440	0.000
PC ae C40:3	1.431	0.003
C14:2	1.390	0.001
C3:1	1.385	0.000
SM C18:1	1.380	0.001

PC ae C36:1	1.362	0.003
PC ae C40:4	1.316	0.000
C5-DC / C6-OH	1.308	0.001
PC ae C30:1	1.306	0.005
PE aa C34:1	1.296	0.000
PC ae C34:0	1.291	0.013
lysoPC a C24:0	1.284	0.019
C5:1-DC	1.280	0.000
PS aa C40:3	1.273	0.013
SM C16:1	1.271	0.001
PC aa C38:3	1.253	0.000
PC ae C38:4	1.251	0.002
C10:1	1.250	0.005
PC ae C34:1	1.246	0.012
PC aa C36:1	1.241	0.018
PE aa C34:0	1.236	0.000
C12:1	1.236	0.001
PC aa C42:2	1.231	0.000
C6 / C4:1-DC	1.227	0.000
PS aa C40:4	1.198	0.004
C18:1	1.194	0.000
PC ae C30:0	1.190	0.002
PC ae C40:5	1.190	0.000
SM C26:1	1.188	0.041
PC ae C38:3	1.184	0.000
PC ae C32:2	1.176	0.002
C3-DC / C4-OH	1.175	0.003
C18:2	1.173	0.000
C10	1.169	0.000
PEA	1.162	0.038
C8	1.153	0.000
C10:2	1.150	0.001
C18:1-OH	1.147	0.000
PC aa C42:6	1.136	0.000
PC aa C32:1	1.122	0.008
PC ae C36:0	1.113	0.000
lysoPE a C18:0	1.095	0.014
PS aa C34:1	1.093	0.000
PC ae C44:4	1.088	0.000
C12-DC	1.082	0.000
C9	1.081	0.002
PC aa C36:6	1.081	0.000
C5-1	1.078	0.002
C6-1	1.071	0.000
PC aa C32:3	1.066	0.000
PC aa C40:1	1.065	0.000
PC ae C40:6	1.062	0.000
C16:1	1.058	0.000
C7-DC	1.045	0.001
PC ae C32:1	1.034	0.012
Orn	1.033	0.000
C14 1	1.015	0.002
C5-OH / C3-DC-M	1.002	0.000
Arg	−1.159	0.005
C16:0 Dihydroceramide	−1.793	0.001
Ser	−2.094	0.001
H1	−2.483	0.000

Table S3. Significant metabolite changes with an absolute $\log_2$ fold change > 1, during dendritic cell differentiation. Given are the $\log_2$ fold changes, compared to the progenitor population and the corresponding FDR adjusted *p*-value.

Metabolite	logFC	adj. p
Histamine	2.527	0.000
PC ae C30:1	2.309	0.001
C3:1	2.268	0.001
SM-OH C14:1	2.189	0.004
C5-DC C6-OH	2.177	0.004
lysoPC a C28:0	2.149	0.001
C6 / C4-1-DC	2.140	0.013
SM-OH C16:1	2.116	0.004
PC ae C32:2	2.026	0.001
C8	1.996	0.007
C18:1 Ceramide	1.975	0.000
PC ae C34:1	1.970	0.008
PC ae C32:1	1.961	0.004
Taurine	1.934	0.001
C14:1	1.915	0.003
PC ae C30:2	1.899	0.000
C6:1	1.889	0.015
C10	1.883	0.005
PC ae C34:2	1.868	0.000
C18:2	1.858	0.005
PC ae C36:3	1.812	0.000
C3-DC / C4-OH	1.797	0.004
PC ae C34:3	1.768	0.000
C7-DC	1.759	0.010
C18:0 Ceramide	1.744	0.000
C16:0 Ceramide	1.728	0.000
C9	1.724	0.002
lysoPC a C28:1	1.705	0.003
C10:1	1.679	0.000
C14:2-OH	1.669	0.003
PC ae C40:3	1.652	0.004
C5-1-DC	1.639	0.012
PC ae C30:0	1.625	0.005
SM C16:0	1.621	0.014
PC ae C38:1	1.615	0.007
C5:1	1.610	0.003
C12:1	1.591	0.000
C4:1	1.585	0.005
C18	1.579	0.044
C4	1.572	0.017
PC aa C28:1	1.566	0.005
PC ae C36:2	1.539	0.000
PC ae C40:5	1.519	0.000
PC ae C38:4	1.498	0.005
C18:1	1.467	0.001
C10:2	1.460	0.000
C14	1.438	0.017
PC ae C40:2	1.399	0.013
C16:2	1.394	0.001
C14:2	1.376	0.004
C16:2-OH	1.372	0.001
C16:1-OH	1.366	0.003
C3-OH	1.359	0.001
PC ae C40:4	1.355	0.002
C3	1.329	0.016
C5	1.326	0.015
PC ae C40:6	1.309	0.001
lysoPC a C20:4	1.308	0.005
C18:1-OH	1.271	0.001
PC ae C36:4	1.271	0.013
C16-OH	1.258	0.005
PC ae C36:1	1.236	0.026
C12	1.225	0.003
PC aa C30:1	1.211	0.000
PC ae C38:6	1.196	0.002
PC ae C32:0	1.187	0.003

C22:0 Ceramide	1.185	0.008
lysoPC a C16:1	1.179	0.004
PC ae C34:0	1.177	0.048
C2	1.151	0.016
SM C18:0	1.143	0.048
PC ae C44:5	1.106	0.000
LPE a C22:4	1.104	0.000
PEA	1.085	0.000
PC ae C44:6	1.078	0.000
C5-OH / C3-DC-M	1.051	0.000
PC aa C38:0	1.049	0.004
PS aa C36:2	1.045	0.000
C14:1-OH	1.026	0.020
SM C26:0	1.023	0.031
C16:1	1.013	0.000
C12-DC	1.001	0.000
PC aa C42:4	−1.005	0.017
PE aa C36:4	−1.061	0.000
PE aa C36:5	−1.063	0.000
PE aa C34:3	−1.086	0.000
PC aa C36:4	−1.123	0.007
PE aa C38:7	−1.129	0.001
Arg	−1.342	0.001
PC aa C38:5	−1.470	0.000
PC aa C36:5	−1.471	0.000
Ser	−1.547	0.006
PE ae C40:1	−1.548	0.000
PE aa C40:7	−1.552	0.000
PE aa C40:6	−1.557	0.000
PC aa C38:6	−1.656	0.000
PE aa C38:6	−1.750	0.000
H1	−1.864	0.000
PE aa C38:5	−2.021	0.000

Table S4. Significant metabolite changes with an absolute $\log_2$ fold change > 1, during neutrophil differentiation. Given are the $\log_2$ fold changes, compared to the progenitor population and the corresponding FDR adjusted *p*-value.

Metabolite	logFC	adj. p
Histamine	8.690	0.000
Taurine	5.277	0.000
SM -OH C14:1	2.821	0.001
C2	2.498	0.000
PC ae C42:4	2.373	0.006
SM-OH C22:1	2.283	0.004
SM-OH C22:2	2.256	0.004
PC ae C38:6	2.114	0.000
PC aa C38:0	1.858	0.001
PC ae C40:6	1.793	0.000
LPE a C22:6	1.702	0.000
SM-OH C16:1	1.483	0.043
C3	1.474	0.014
SM-OH C24:1	1.408	0.039
PC ae C44:6	1.307	0.008
PC ae C44:5	1.218	0.017
PC aa C42:0	1.199	0.000
PC ae C44:4	1.065	0.007
PC aa C38:6	1.009	0.014
PE aa C38:4	−1.000	0.000
PS aa C34:1	−1.011	0.000
PC aa C40:4	−1.015	0.048
PC ae C42:1	−1.127	0.001
PE aa C36:2	−1.129	0.000
PC aa C32:1	−1.161	0.048
PE aa C36:1	−1.170	0.000
PE ae C42:1	−1.199	0.008
PE aa C34:0	−1.263	0.000
PE aa C40:3	−1.278	0.000
PE aa C38:5	−1.518	0.000
PC aa C32:3	−1.539	0.001

Gly	−1.545	0.014
PC aa C32:2	−1.553	0.004
SM C22:3	−1.562	0.041
PS aa C40:4	−1.563	0.001
PC aa C36:4	−1.583	0.012
PE aa C34:2	−1.660	0.000
PC aa C30:0	−1.644	0.021
lysoPC a C18:2	−1.662	0.002
PE aa C40:5	−1.712	0.000
PC aa C36:3	−1.713	0.001
PE aa C34:3	−1.719	0.000
Phe	−1.719	0.000
Val	−1.785	0.038
PC aa C34:4	−1.791	0.000
Tyr	−1.796	0.001
PE aa C34:1	−1.815	0.000
H1	−1.839	0.000
Ile	−1.896	0.002
Glu	−1.902	0.013
PC aa C34:2	−1.904	0.001
PE aa C36:4	−1.949	0.000
Leu	−1.964	0.004
Met	−1.978	0.002
PC aa C36:2	−1.984	0.001
Thr	−2.002	0.012
Asp	−2.008	0.014
SM C20:2	−2.111	0.001
PE aa C40:4	−2.129	0.000
C24:1 Ceramide	−2.182	0.000
C16:0 Ceramide	−2.264	0.000
PC aa C34:3	−2.269	0.000
PE aa C36:3	−2.461	0.000
Pro	−2.692	0.000
Lys	−2.823	0.000
Ser	−2.841	0.000
C18:0 Ceramide	−2.995	0.000
His	−3.057	0.001
Asn	−3.175	0.000
Ala	−3.363	0.000
Arg	−3.836	0.000
C22:0 Ceramide	−4.024	0.000
C16:0 Dihydroceramide	−4.811	0.000

Table S5. Unique metabolite changes during erythroid differentiation. Given are the $\log_2$ fold changes, compared to the progenitor population and the corresponding FDR adjusted *p*-value.

Metabolite	logFC	adj. p
Putrescine	1.495	0.000
lysoPC a C24:0	1.284	0.019
PC aa C32:1	1.122	0.008
PC aa C32:3	1.066	0.000
PC aa C38:3	1.253	0.000
PC aa C40:2	1.656	0.000
PC aa C40:3	1.769	0.000
PC aa C40:4	1.566	0.000
PC aa C42:2	1.231	0.000
PC aa C42:4	1.907	0.000
PC aa C42:5	1.611	0.000
PE aa C34:0	1.235	0.000
PE aa C34:1	1.296	0.000
PE aa C40:3	1.553	0.000
PE aa C40:4	1.439	0.000
PS aa C40:3	1.272	0.013
SM C26:1	1.188	0.041

Table S6. Unique metabolite changes during dendritic cell differentiation. Given is the $\log_2$ fold change, compared to the progenitor population and the corresponding FDR adjusted *p*-value.

Metabolite	logFC	adj. p
C16-OH	1.258	0.005
C16:1-OH	1.366	0.003
PC aa C36:5	−1.471	0.000
PC aa C38:6	−1.656	0.000
PC ae C30:1	2.309	0.001
PC ae C34:2	1.868	0.000
PC ae C36:3	1.812	0.000
PE aa C38:6	−1.750	0.000
PE aa C38:7	−1.129	0.001
PE aa C40:6	−1.556	0.000
PE aa C40:7	−1.551	0.000
C16:0 Ceramide	1.727	0.000
C18:0 Ceramide	1.743	0.000
C18:1 Ceramide	1.974	0.000

Table S7. Unique metabolite changes during neutrophil differentiation. Given is the $\log_2$ fold change, compared to the progenitor population and the corresponding FDR adjusted *p*-value.

Metabolite	logFC	adj. p
Ala	−3.363	0.000
Arg	−3.836	0.000
Asn	−3.175	0.000
Asp	−2.008	0.014
Glu	−1.902	0.013
Gly	−1.545	0.014
His	−3.057	0.001
Ile	−1.896	0.002
Leu	−1.964	0.004
Lys	−2.823	0.000
Met	−1.978	0.002
Phe	−1.719	0.000
Pro	−2.692	0.000
Thr	−2.002	0.012
Tyr	−1.796	0.001
Val	−1.785	0.038
Histamine	8.690	0.000
Taurine	5.277	0.000
C2	2.498	0.000
PC aa.C30:0	−1.644	0.021
PC aa.C32:1	−1.161	0.048
PC aa.C32:3	−1.539	0.001
PC aa.C34:2	−1.904	0.001
PC aa.C34:3	−2.269	0.000
PC aa.C34:4	−1.791	0.000
PC aa.C36:2	−1.984	0.001
PC aa.C36:3	−1.713	0.001
PC aa.C38:6	1.009	0.014
lysoPE a C22:6	1.702	0.000
PE aa C34:0	−1.263	0.000
PE aa C34:1	−1.815	0.000
PE aa C36:1	−1.170	0.000
PE aa C36:2	−1.129	0.000
PE aa C36:3	−2.461	0.000
PE aa C40:3	−1.278	0.000
PE aa C40:4	−2.129	0.000
PS aa C34:1	−1.011	0.000
PS aa C40:4	−1.563	0.000
C16:0 Ceramide	−2.264	0.000
C16:0 Dihydroceramide	−4.811	0.000

C18:0 Ceramide	−2.995	0.000
C22:0 Ceramide	−4.024	0.000
C24:1 Ceramide	−2.182	0.000
SM-OH C22:1	2.283	0.004
SM C20:2	−2.111	0.001

Table S8. Common metabolite changes during erythroid and dendritic cell differentiation. Given are the log_2 fold changes in each population, compared to the progenitor population. All changes in the erythroid and the dendritic cell population own an FDR adjusted *p*-value < 0.05.

Metabolite	erythrocytes	dendritic cells	neutrophils
C10	1.883	1.169	−0.348
C10:2	1.460	1.150	−0.181
C12:1	1.591	1.236	0.075
C12-DC	1.001	1.082	−0.087
C14:2	1.376	1.390	0.246
C16:1	1.013	1.058	0.010
C18:1	1.467	1.194	−0.143
C18:1-OH	1.271	1.147	−0.060
C18:2	1.858	1.173	−0.054
C3:1	2.268	1.385	0.014
C3-DC (C4-OH)	1.797	1.175	0.172
C5:1	1.610	1.078	−0.100
C5:1-DC	1.639	1.280	−0.041
C5-DC (C6-OH)	2.177	1.308	0.093
C6 (C4:1-DC)	2.140	1.227	−0.341
C6:1	1.889	1.071	−0.121
C8	1.996	1.153	−0.316
C9	1.724	1.081	0.033
lysoPC a C28:0	2.149	1.681	0.605
PC aa C28:1	1.566	1.723	−0.874
PC ae C30:0	1.625	1.190	−0.127
PC ae C34:1	1.970	1.246	−0.500
PC ae C38:4	1.498	1.251	−0.069
SM C16:0	1.621	1.576	−0.344
SM C18:0	1.143	1.522	−0.519

Table S2. Common metabolite changes during erythroid and neutrophil differentiation. Given are the log_2 fold changes in each population, compared to the progenitor population. All changes in the erythroid and the neutrophil population own an FDR adjusted *p*-value < 0.05.

Metabolite	Erythrocytes	Dendritic Cells	Neutrophils
SM-OH C22:2	1.577	0.298	2.256
C16 Dihydroceramide	−1.793	−0.180	−4.810

Table S3. Common metabolite changes during dendritic cell and neutrophil differentiation. Given are the log_2 fold changes in each population, compared to the progenitor population. All changes in the dendritic cell and the neutrophil population own an FDR adjusted *p*-value < 0.05.

Metabolite	erythrocytes	dendritic cells	neutrophils
Taurine	−0.644	1.934	5.277
PC aa C36:4	0.617	−1.123	−1.583
PE aa C34:3	0.374	−1.086	−1.719
PE aa C36:4	0.995	−1.060	−1.949
PE aa C38:5	−0.407	−2.020	−1.517
SM C22:3	0.763	−1.240	−1.562

Table S11. Common metabolite changes during all differentiations. Given are the $\log_2$ fold changes in each population, compared to the progenitor population. All changes own an FDR adjusted *p*-value < 0.05.

Metabolite	Erythrocytes	Dendritic Cells	Neutrophils
Arg	−1.158	−1.341	−3.836
Ser	−2.093	−1.547	−2.841
Histamine	1.733	2.526	8.690
PC ae C40:6	1.062	1.309	1.793
PC ae C42:4	2.321	3.669	2.373
SM-OH C14:1	1.441	2.188	2.820
SM-OH C16:1	1.801	2.1159	1.483
H1	−2.482	−1.864	−1.839
